# Ectopic Calcification and Hypophosphatemic Rickets: Natural History of ENPP1 and ABCC6 Deficiencies

**DOI:** 10.1002/jbmr.4418

**Published:** 2021-08-16

**Authors:** Carlos R Ferreira, Kristina Kintzinger, Mary E Hackbarth, Ulrike Botschen, Yvonne Nitschke, M Zulf Mughal, Genevieve Baujat, Dirk Schnabel, Eric Yuen, William A Gahl, Rachel I Gafni, Qing Liu, Pedro Huertas, Gus Khursigara, Frank Rutsch

**Affiliations:** ^1^ National Human Genome Research Institute National Institutes of Health Bethesda MD USA; ^2^ Department of General Pediatrics Münster University Children's Hospital Münster Germany; ^3^ Department of Paediatric Endocrinology Royal Manchester Children's Hospital Manchester UK; ^4^ Centre de Référence Maladies Osseuses Constitutionnelles (CR MOC) et Filière OSCAR, Département de Génétique Hôpital Necker‐Enfants Malades Paris France; ^5^ Center for Chronically Sick Children, Pediatric Endocrinology Charité‐Universitätsmedizin Berlin Berlin Germany; ^6^ Inozyme Pharma, Inc. Boston MA USA; ^7^ National Institute of Dental and Craniofacial Research National Institutes of Health Bethesda MD USA; ^8^ Quantitative & Regulatory Medical Science, LLC Long Valley NJ USA

**Keywords:** ABCC6 deficiency, diseases and disorders of/ related to bone, ectopic calcification, ectopic calcification, ENPP1 deficiency, Generalized Arterial Calcification of Infancy, hypophosphatemic rickets, natural history study, osteomalacia, rickets, survival analysis

## Abstract

Generalized arterial calcification of infancy (GACI) is a rare disorder caused by *ENPP1* or *ABCC6* variants. GACI is characterized by low pyrophosphate, arterial calcification, and high mortality during the first year of life, but the natural course and possible differences between the causative genes remain unknown. In all, 247 individual records for patients with GACI (from birth to 58.3 years of age) across 19 countries were reviewed. Overall mortality was 54.7% (13.4% in utero or stillborn), with a 50.4% probability of death before the age of 6 months (critical period). Contrary to previous publications, we found that bisphosphonate treatment had no survival benefit based on a start‐time matched analysis and inconclusive results when initiated within 2 weeks of birth. Despite a similar prevalence of GACI phenotypes between ENPP1 and ABCC6 deficiencies, including arterial calcification (77.2% and 89.5%, respectively), organ calcification (65.8% and 84.2%, respectively), and cardiovascular complications (58.4% and 78.9%, respectively), mortality was higher for *ENPP1* versus *ABCC6* variants (40.5% versus 10.5%, respectively; *p* = 0.0157). Higher prevalence of rickets was reported in 70.8% of surviving affected individuals with *ENPP1* compared with that of *ABCC6* (11.8%; *p* = 0.0001). Eleven affected individuals presenting with rickets and without a GACI diagnosis, termed autosomal recessive hypophosphatemic rickets type 2 (ARHR2), all had confirmed *ENPP1* variants. Approximately 70% of these patients demonstrated evidence of ectopic calcification or complications similar to those seen in individuals with GACI, which shows that ARHR2 is not a distinct condition from GACI but represents part of the spectrum of ENPP1 deficiency. Overall, this study identified an early mortality risk in GACI patients despite attempts to treat with bisphosphonates, high prevalence of rickets almost exclusive to ENPP1 deficiency, and a spectrum of heterogenous calcification and multiple organ complications with both *ENPP1* and *ABCC6* variants, which suggests an overlapping pathology. © 2021 The Authors. *Journal of Bone and Mineral Research* published by Wiley Periodicals LLC on behalf of American Society for Bone and Mineral Research (ASBMR). This article has been contributed to by US Government employees and their work is in the public domain in the USA.

## Introduction

Generalized arterial calcification of infancy (GACI) is a rare autosomal recessive disorder characterized by calcification of arteries and marked neointimal proliferation leading to arterial stenoses and cardiac complications.^(^ ^1^ ^)^ Individuals with GACI also develop calcification in joints and parenchymal organs and demonstrate renal, gastrointestinal, and pulmonary complications.^(^ ^2^, ^3^ ^)^ The disease can manifest prenatally and has a mortality of ~55% in the first 6 months of life despite intensive therapy (critical mortality period).^(^ ^4^ ^)^ However, the disease course after the initial 6 months is not well characterized, with reports limited to case series or small retrospective studies.^(^ ^4^, ^5^, ^6^ ^)^

Biallelic inactivating variants in *ENPP1* (ectonucleotide pyrophosphatase/phosphodiesterase 1) or *ABCC6* (ATP binding cassette subfamily C member 6) account for ~75% and 9% of GACI cases, respectively.^(^ ^4^, ^7^ ^)^ ENPP1 is an integral transmembrane protein that hydrolyzes extracellular adenosine triphosphate (ATP) to inorganic pyrophosphate (PP_i_) and adenosine monophosphate (AMP). PP_i_ is a potent inhibitor of calcium hydroxyapatite crystal deposition and is essential to prevent ectopic soft tissue calcification.^(^ ^8^ ^)^ AMP is further hydrolyzed to adenosine, a potent inhibitor of neointimal proliferation.^(^ ^9^ ^)^ Vascular calcification and arterial stenosis are thought to occur as a result of the reduction in PP_i_ and AMP, respectively.^(^ ^7^, ^10^, ^11^ ^)^ *Enpp1* knockout mice show a similar phenotype to patients diagnosed with GACI: low serum PP_i_, extensive arterial calcification, arterial stenosis, and calcification of selected organs.^(^ ^7^, ^11^ ^)^ Although the exact role of ABCC6 in GACI is not well understood, reduction in serum PP_i_ levels and ectopic calcifications are also observed in mouse models and patients with *ABCC6* variants, which suggests a common PP_i_ pathway between ENPP1 and ABCC6 deficiencies.^(^ ^12^, ^13^ ^)^ However, it is not clear if the clinical profile and disease course are similar between *ABCC6* and *ENPP1* variants.

Due to low PP_i_ and extensive calcification in GACI patients, it stands to reason that PP_i_ analogues such as bisphosphonates are often used as treatment to attempt to reduce mortality. However, the benefit of using bisphosphonates for GACI patients is not clear.^(^ ^4^, ^5^, ^6^ ^)^

Individuals with GACI who survive infancy have been reported to progress to autosomal recessive hypophosphatemic rickets type 2 (ARHR2).^(^ ^14^, ^15^ ^)^ ARHR2 manifests clinically as short stature and bone deformities and is associated with *ENPP1* variants, but it has not been reported to be associated with *ABCC6* variants.^(^ ^16^, ^17^, ^18^, ^19^, ^20^ ^)^ GACI survivors have been found to have elevated fibroblast growth factor 23 (FGF23), and this may contribute to the hypophosphatemic rickets and osteomalacia.^(^ ^4^, ^16^, ^17^ ^)^

A genotypic and phenotypic overlap of GACI with pseudoxanthoma elasticum (PXE) has been reported.^(^ ^7^ ^)^ Classic PXE is caused by *ABCC6* variants, manifests as soon as the second decade of life with skin changes, and is characterized by mineralization and fragmentation of elastic fibers in skin, eyes, and cardiovascular system.^(^ ^19^, ^20^ ^)^ There are case reports of individuals diagnosed with GACI due to *ENPP1* variants who developed PXE‐like symptoms, which suggests that PXE‐like symptoms may not be limited to genotype but to a common mechanism of low PP_i_ and ectopic calcification.^(^ ^7^ ^)^

The natural history of GACI, in particular its mortality, relation to rickets, and phenotypic differences based on genetic loci, has not been comprehensively elucidated, limiting effective diagnosis, prognosis, and management.^(^ ^4^, ^7^ ^)^ This study describes the natural history of GACI, role of bisphosphonates in survival, and phenotypic similarities/differences between ENPP1 and ABCC6 deficiencies.

## Patients and Methods

### Study design and patients

This retrospective international cross‐sectional study included individuals with GACI or ARHR2 confirmed by imaging, biopsy, biochemical findings, and/or *ENPP1* or *ABCC6* variants or by parental mutational analysis indicating *ENPP1*/*ABCC6* variants consistent with symptoms of affected individuals. No exclusion criteria were implemented. Data were compiled from two primary studies by the US National Institutes of Health (NIH) (NCT03478839) and Germany's Münster University Children's Hospital (UKM) (NCT03758534). Data from cases diagnosed between 1960 and 2018 (median, 2010) were retrieved between 2018 and 2020. The study was conducted in accordance with the Declaration of Helsinki and with approval from local institutional review board/ethics commissions prior to data collection. Data from living affected individuals or legal guardians of minors were collected after obtaining consent. For deceased affected individuals, data were collected from legal guardians or physicians.

### Procedures

Data were extracted retrospectively from medical chart reviews to develop case report forms (CRFs); CRFs for the two primary study sites were almost identical, allowing data combination. The CRFs included details on vital status, diagnosis, mutational analysis, medical/birth histories, initial postnatal GACI presentation, clinical course, treatments, and laboratory results—the full data set. When data were available but no consent was obtained from affected individuals/legal guardians (eg, deceased individuals), or when physicians were unwilling to participate, a five‐item questionnaire was used. Information on vital status (if alive, current age; if deceased, age at death), history of bisphosphonates, and rickets or PXE diagnosis was collected for the minimal data set. A greater nonparticipation rate among physicians of deceased affected individuals was anticipated; this could result in biased overestimate of survival. Thus, the minimal data set was included to mitigate this bias and to obtain accurate survival estimate.

### Data collection and management

The identity of affected individuals remained confidential during data transfer. In the NIH study, medical records were collected and reviewed by site staff, and extracted data were entered directly into an electronic data capture system, exported, and transferred securely. Source data verification of key information was conducted. In the UKM study, the treating physician extracted the data from medical charts and entered the data directly into an electronic CRF (eCRF). Any inconsistent data were clarified via physician consultation; duplicate data sets were removed. Data were combined using R 9.4 (R Foundation for Statistical Computing, Vienna, Austria; https://www.r-project.org/).

### Outcomes

The primary objective was to characterize survival. Secondary objectives were to characterize natural history of GACI, elucidate role of bisphosphonates in survival, and assess clinical differences between ENPP1 and ABCC6 deficiencies.

### Statistical analysis

Descriptive statistics were calculated to summarize data. Survival rates were determined using Kaplan‐Meier analysis. Age at death or age last known alive was used for survival calculation; where only month and year of birth and death were available, the day was imputed to the first of the month for birth and last of the month for death. For individuals who died in utero or were stillborn, the age at death was set to 0. In the minimal data set, two affected individuals were excluded because age at death was not specified (*n* = 1) and age at last follow‐up was unavailable (*n* = 1); age at death or last known alive was imputed for nine affected individuals. In the full data set, dates of birth (*n* = 69) and death (*n* = 8), and ages of affected individuals who died in utero or were stillborn (*n* = 10), were imputed as described. An affected individual start‐time matched analysis was used to assess the outcome of bisphosphonate treatment on survival. Statistical analyses were performed using SAS 9.4 (SAS Institute Inc., Cary, NC, USA) and RStudio 1.2.1335 (RStudio; https://www.rstudio.com/).

## Results

The data set (*N* = 247) included 127 affected individuals in the full data set (56 from NIH and 71 from UKM) and 120 affected individuals in the minimal data set (eight from NIH and 112 from UKM). The data from the five‐item questionnaire for the minimal data set are included in Table 1.

**Table 1:** Diagnosis, Bisphosphonate Treatment, and Vital Status

Parameter	All affected individuals (*N* = 247)	Minimal data set (*n* = 120)	Full data set (*n* = 127)	ENPP1 data set (*n* = 84)^a^	ABCC6 data set (*n* = 19)^a^
Follow‐up age (months), mean (range)	53.2 (0.0–709.7)	28.7 (0.0–420.3)	27.4 (0.0–709.7)	34.4 (0.0–709.7)	83.2 (0.1–154.4)
Gender, female/male (% female)	–	–	59/67 (46.8)	41/42 (49.4)	7/12 (36.8)
GACI					
Yes/no (% yes of assessed)			115/11 (91.3)	73/11 (86.9)	18/0 (100)
Age at diagnosis (months), median (range)^b^			0.8 (−4.7 to 137.0)	0.7 (−4.7 to 137.0)	3.0 (−3.1 to 73.4)
Rickets					
Yes/no (% yes of assessed)	45/164 (27.4)	5/95 (5.0)	40/69 (36.7)	36/38 (48.6)	3/18 (16.7)
Age at diagnosis (months), median (range)^b^	52.9 (0.5–348.0)	204 (48.0–348.0)	38.3 (0.5–222.1)	52.9 (0.5–222.1)	27.5 (8.2–46.7)
PXE					
Yes/no (% yes of assessed)	9/197 (4.4)	1/99 (1.0)	8/98 (7.5)	5/63 (7.4)	3/13 (18.8)
Age at diagnosis (months), median (range)^b^	49.7 (2.7–524.0)	8.0 (8.0–8.0)	72.7 (2.7–524)	115.0 (26.6–524.0)	4.5 (2.7–6.2)
Bisphosphonate treatment					
Yes/no (% yes of assessed)	93/116 (45.5)	30/65 (46.2)	63/51 (55.3)	45/30 (60.0)	13/3 (68.4)
Alive, *n* (%)	112 (45.3)	43 (36.4)	68 (53.5)	50 (59.5)	17 (89.5)
Last known age (months), median (range)^b^	62.8 (0.0–709.7)	16.0 (0.0–420.3)	98 (3.2–709.7)	111.1 (3.2–709.7)	90.4 (4.8–154.4)
Deceased, *n* (%)	135 (54.7)	75 (63.5)	59 (46.5)	34 (40.5)	2 (10.5)
Age at death (months), median (range)^b^ ^,^ ^c^	0.8 (0.0–182.5)	0.2 (0.0–182.5)	1.2 (0.0–127.0)	2.0 (0.0–61.8)	3.9 (0.1–7.7)
Prenatal death (%)	13.30	19.20	7.90	4.70	5.20
Deceased in utero, *n*	21	14	7	3	1
Stillborn, *n*	12	9	3	1	0

GACI = generalized arterial calcification of infancy; PXE = pseudoxanthoma elasticum.

^a^ Isolated variant: does not include 3 affected individuals (all 3 deceased) with both *ENPP1* and *ABCC6* variants.

^b^ Based on available dates.

^c^ For those who were stillborn or died in utero, the age at death was set to 0 months.

### Demographics

**Fig 1:**
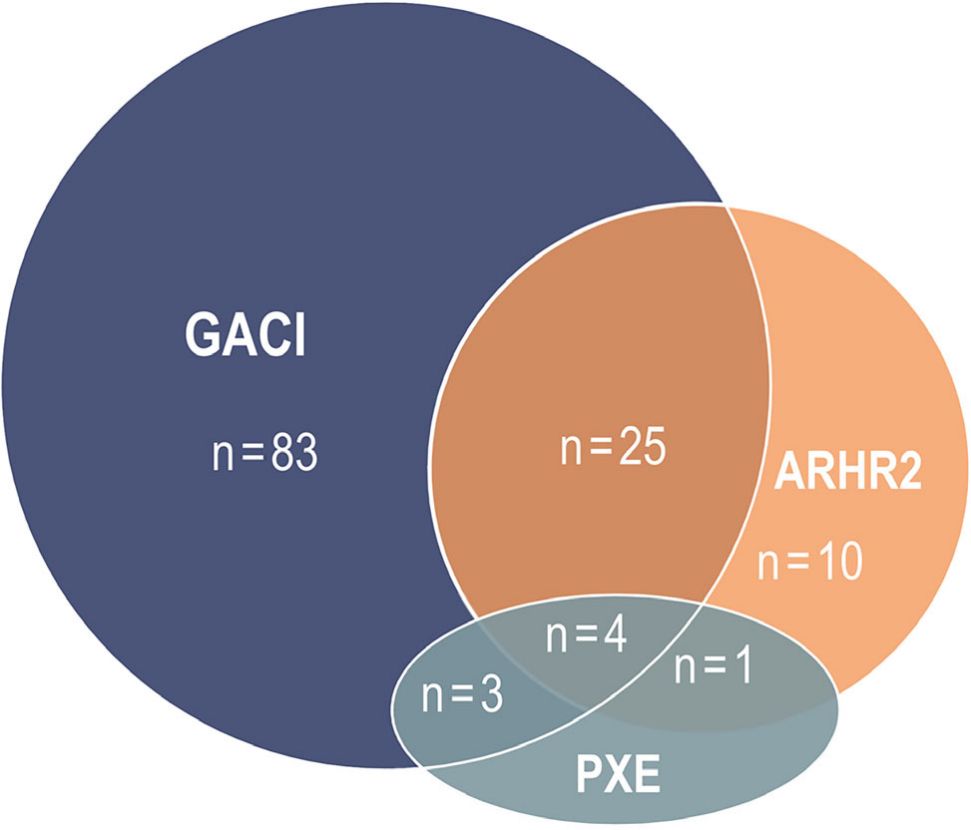
Overlap of GACI, rickets, and PXE diagnoses in affected individuals in the full data set (*n* = 126). One individual manifested only widespread arterial stenoses, but not calcification, rickets, or PXE‐like changes, and thus was not included. ARHR2 = autosomal recessive hypophosphatemic rickets type 2; GACI = generalized arterial calcification of infancy; PXE = pseudoxanthoma elasticum.

Of the affected individuals in the full data set (*n* = 127), 52.8% were male, 46.5% were female, and sex was unknown in one individual. Consanguinity of biological parents was documented in 27 cases (21.3%) and unknown in 37 (29.1%). Diagnoses of GACI, rickets, and PXE were documented in 115 (91.3%), 40 (36.7%), and eight (7.5%), respectively (Table 1). Overlap of multiple diagnoses was observed (Fig. 1). Of the affected individuals who survived the first day of life (*n* = 117), 96 (82.1%) were hospitalized at initial presentation due to severity of their symptoms. Mutational analysis was performed for 116 affected individuals and 76 (65.0%) parents in the full data set. A total of 84 of 127 affected individuals (66.1%) had confirmed isolated *ENPP1* variants according to direct mutational analyses, 19 affected individuals (15.0%) had variants in *ABCC6*, three (2.4%) had both *ENPP1* and *ABCC6* variants, and 21 affected individuals (16.5%) did not harbor (or were unknown to harbor) any *ENPP1* or *ABCC6* variants. In three affected individuals who had mutations in both genes, mutations were biallelic for *ENPP1* and monoallelic for *ABCC6*. Diagnosis of GACI, rickets, and PXE in affected individuals with isolated *ENPP1* and *ABCC6* variants is presented in Table 1.

### Mortality

The overall mortality of all affected individuals (*N* = 247) was 54.7%, of which 33 died in utero or were stillborn, accounting for 24.4% of total deaths (Table 1). Mortality was higher in the minimal data set (63.5%) compared with that in the full data set (46.5%), and median age at death was lower in the minimal data set (0.2 months) compared with that in the full data set (1.2 months). Of the affected individuals in the full data set with a recorded cause of death (*n* = 52), cardiovascular complications (cardiac arrest, heart failure, myocardial infarction) were most prevalent (32.7%).

**Fig 2:**
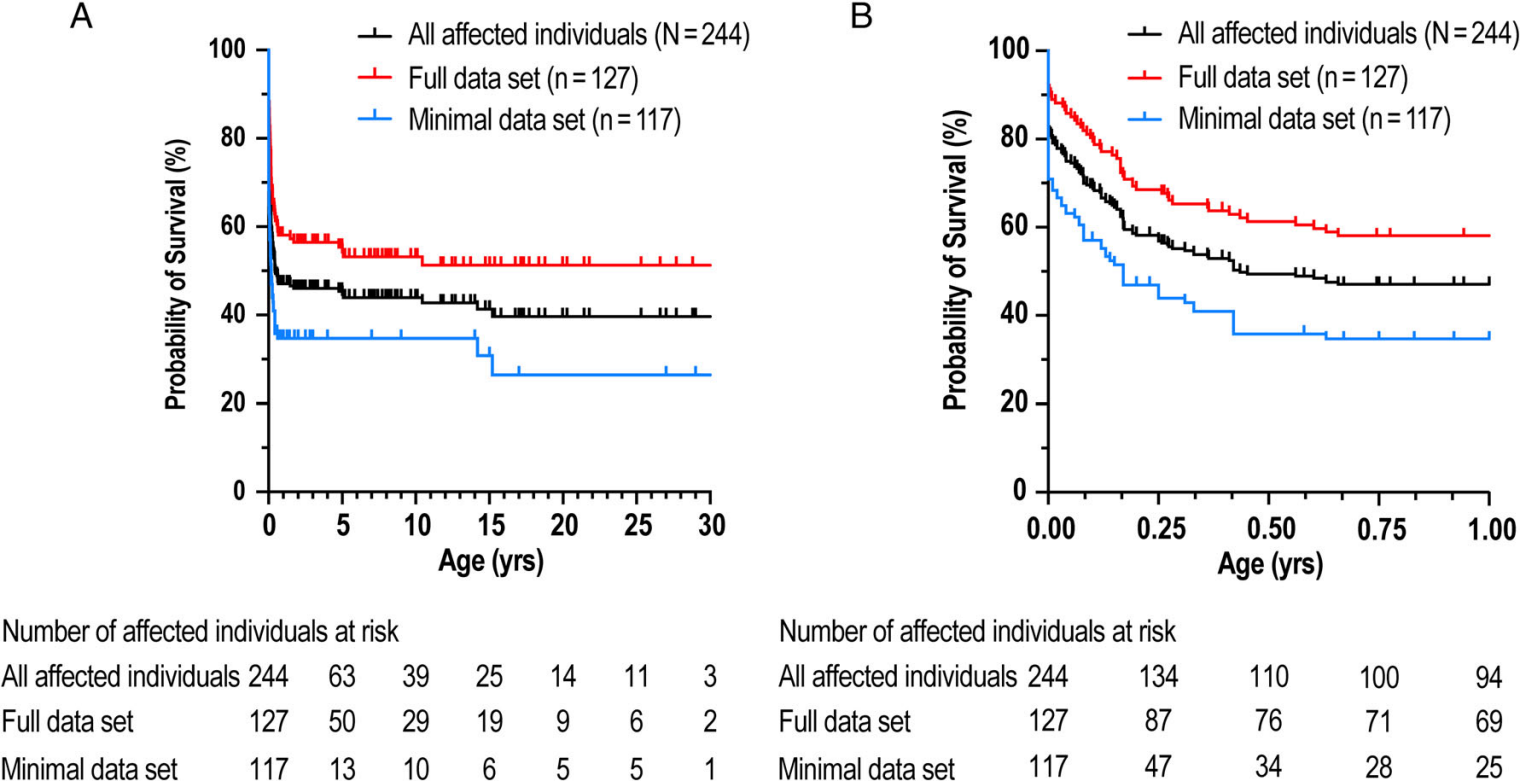
Survival analysis. Kaplan‐Meier estimates of survival in all, full, and minimal data sets at (*A*) 0–30 years and (*B*) 0–1 year.

**Fig 3:**
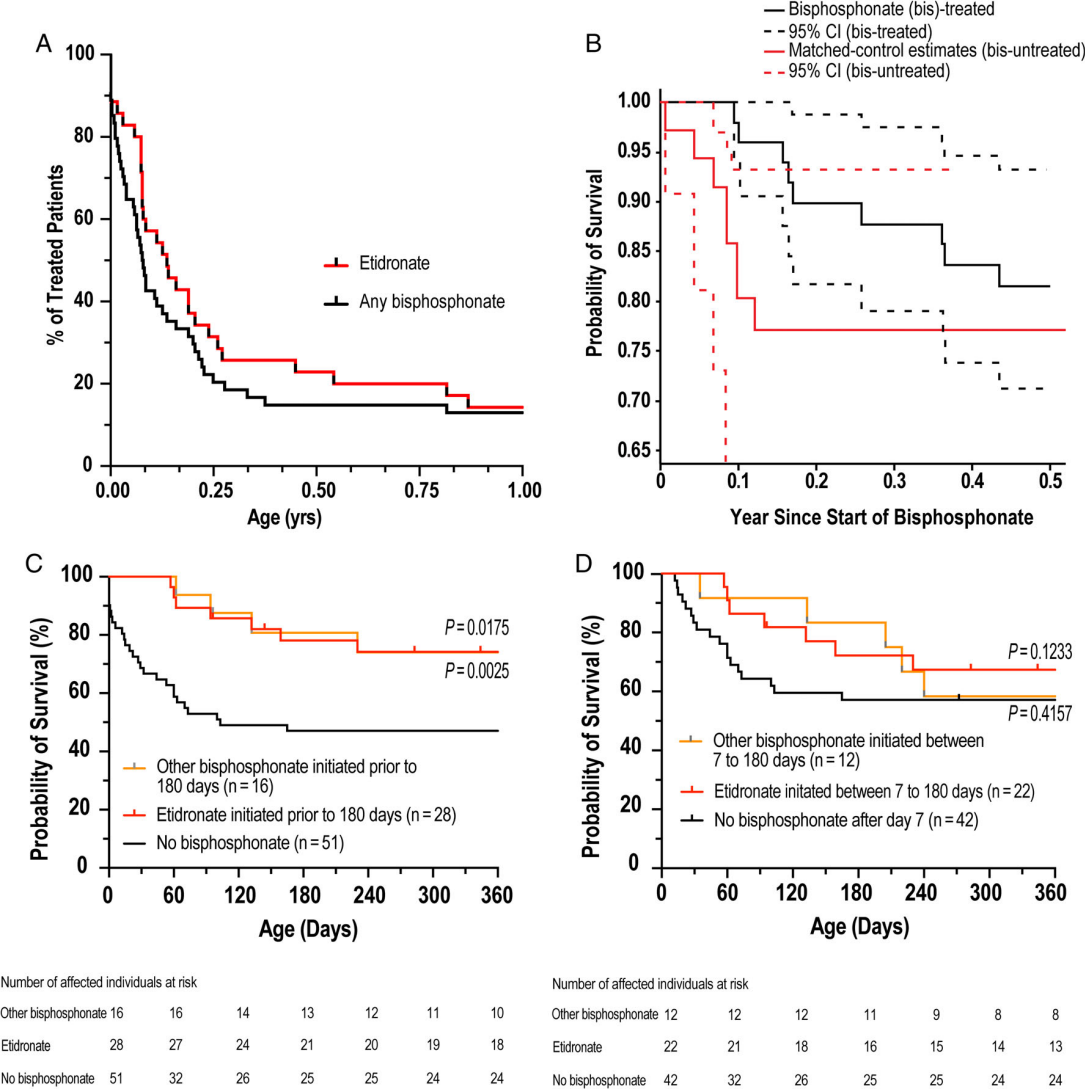
(*A*) Time to bisphosphonate initiation and (*B*) affected individual‐matched KM survival analysis based on bisphosphonate treatment. KM survival estimate for etidronate initiated (*C*) prenatally or (*D*) from age of day 7. KM = Kaplan‐Meier.

Kaplan‐Meier survival estimates demonstrated high risk of mortality within the first 6 months of life and became stable with increasing age (Fig. 2*A*,*B*). The probability of mortality at 6 months was 50.6% for the total population (38.8% and 64.1% in full and minimal data sets, respectively). In the full data set, 55.2% (63/114 assessed) were treated with bisphosphonates (Table 2). A breakdown of the top three used bisphosphonates is presented in Table 2 including patients that used multiple agents. There were a number of missing doses, wide range of doses and inconsistent delivery, or frequency not reported. Initiation of bisphosphonate varied during the critical period of the first 6 months (Fig. 3*A*). Delay in initiating bisphosphonate after the first few weeks or months when mortality risk is lower than at birth might offer affected individuals a survival bias with respect to bisphosphonate treatment. In order to assess if treatment with bisphosphonate would offer a survival advantage, we focused on the first 6 months of age where the risk of mortality is highest. An affected individual start‐time matched estimate was used to compare the risk of mortality in affected individuals. In short, each affected individual who initiated bisphosphonate prior to 6 months of age was matched by age to affected individuals not receiving bisphosphonate, and risk of mortality from that age forward was compared. Because of the small number of etidronate‐treated affected individuals for a matched analysis, all bisphosphonate classes were included. There was no statistically significant survival benefit between individuals treated with bisphosphonate and those untreated (Fig. 3*B*). The confidence intervals overlapped after the first month of life, which suggests inconclusive benefit of early treatment within 1 month of age. Additionally, a Kaplan‐Meier survival analysis was performed focused on affected individuals who initiated etidronate within 6 months of age, other bisphosphonates (no etidronate) initiated within 6 months of age, and affected individuals who did not receive bisphosphonates. The analysis demonstrated significant survival benefit for either etidronate or other bisphosphonates versus untreated at 1 year (74%, 74% versus 47%, respectively; log‐rank [Cox‐Mantel] test *p* = 0.0025 and *p* = 0.0175) (Fig. 3*C*); however, there was no statistical difference in survival if etidronate or any other bisphosphonate was initiated 7 days or later after birth (*p* =0.1233, *p* = 0.4157, respectively) (Fig. 3*D*).

**Table 2:** Bisphosphonate Use in the Full Data Set

Parameter	Use of bisphosphonate, yes/assessed (% yes)	Age at initiation, median month (range)
Full data set (*n* = 127)		
All bisphosphonates	63/114 (55.2)	
Etidronate	42/63 (66.7)	1.6 (−3.1 to 342)
Pamidronate	29/63 (46)	0.8 (0.0–70.7)
Risedronate	9/63 (14.2)	2.6 (0.6–66.7)
Multiple agents	40/63 (63.5)	
ENPP1 (*n* = 84)		
All bisphosphonates	45/75 (60.0)	
Etidronate	27/45 (60.0)	1.2 (−3.1 to 342.0)
Pamidronate	22/45 (48.9)	0.7 (0.0–70.7)
Risedronate	8/45 (17.8)	2.0 (0.6–66.7)
Multiple agents	29/45 (64.4)	
ABCC6 (*n* = 19)		
All bisphosphonates	13/19 (68.4)	
Etidronate	13/13 (100.0)	5.5 (−1.6 to 62.5)
Pamidronate	5/13 (38.5)	2.0 (0.7–3.4)
Risedronate	0/13 (0.0)	
Multiple agents	9/13 (69.2)	

### Phenotype in ENPP1 versus ABCC6 deficiencies

**Fig 4:**
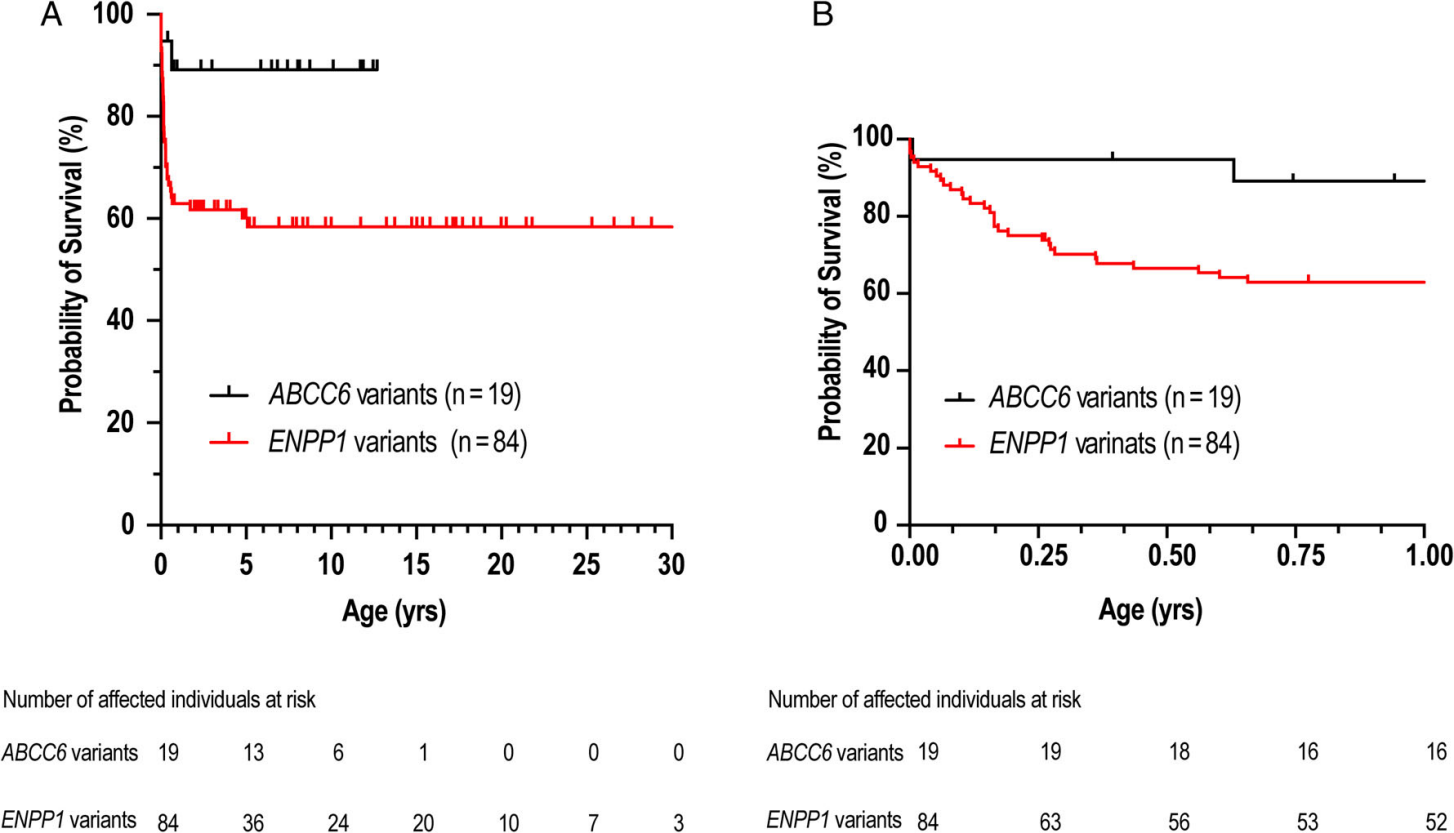
Survival analysis. Kaplan‐Meier estimates of survival in ENPP1‐deficient and ABCC6‐deficient affected individuals aged (*A*) 0 to 30 years and (*B*) 0 to 1 year.

The risk of mortality by 6 months and 12 months was significantly higher in affected individuals with *ENPP1* (*n* = 84; 33.5%, 37%, respectively) versus *ABCC6* variants (*n* = 19; 5.3%, 10.8%, respectively) (log‐rank [Cox‐Mantel] test *p* =0.0214) (Fig. 4*A*,*B*). The 21 affected individuals with no identified sequence variants in *ENPP1* or *ABCC6* had a higher mortality rate (probability of mortality at 6 months: 95%) than the other two cohorts. Of the 21 affected individuals, mutational analysis was performed on seven affected individuals but no variants were reported. Six affected individuals had mother and father with identified *ENPP1* variants, but no affected individuals had parents with identified *ABCC6* variants. When the six affected individuals with parents carrying an *ENPP1* variant are included in the ENPP1 cohort, the 6‐month and 12‐month risk of mortality was 36.8% and 41.3%, respectively.

**Fig 5:**
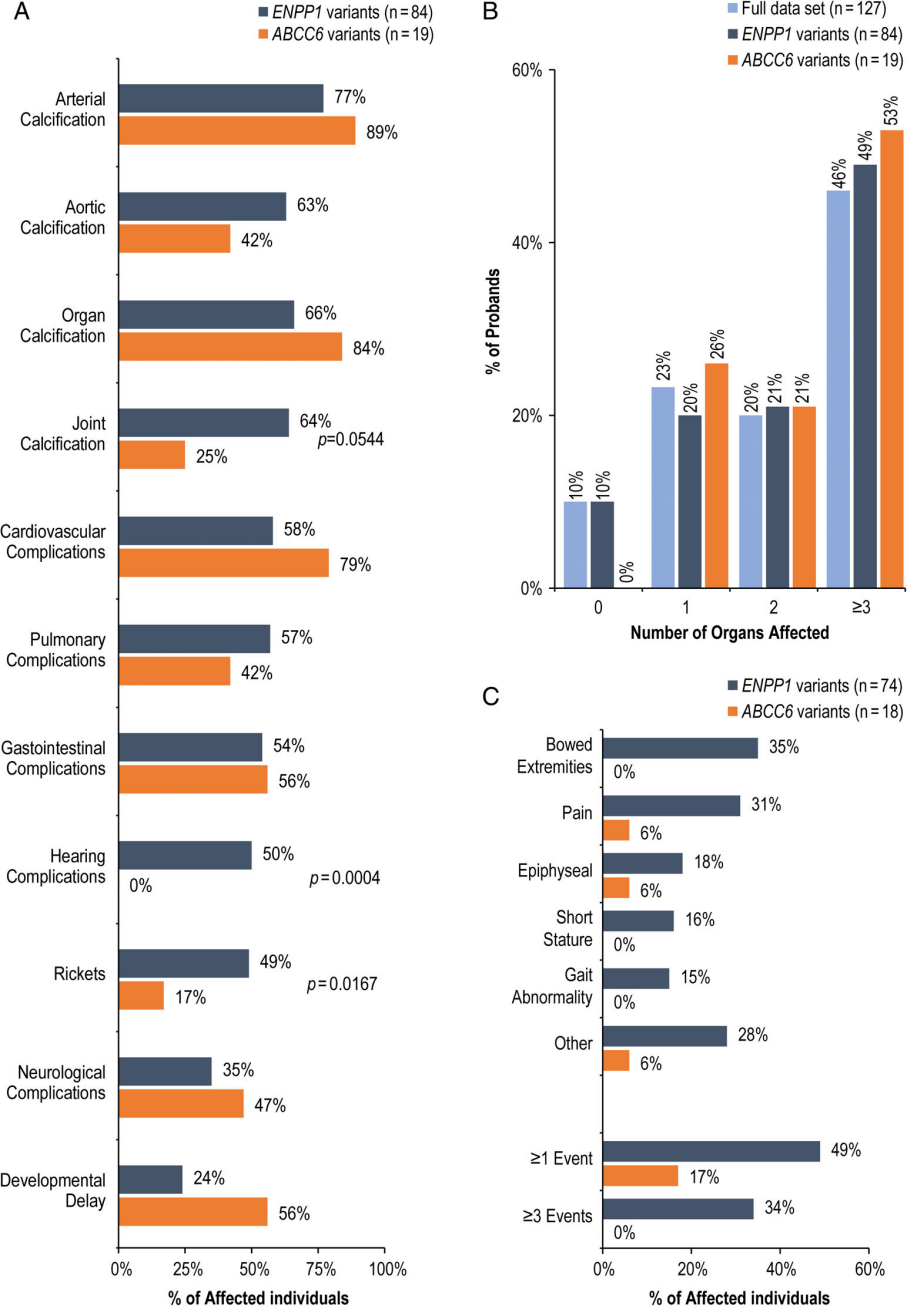
(*A*) Prevalence of calcification and organ complications in affected individuals with *ENPP1* and *ABCC6* variants. (*B*) Affected individuals with multiple organ complications. (*C*) Prevalence of rickets signs/symptoms. The percentages were calculated based on the number of affected individuals who were evaluated for manifestation.

Prevalence of calcification of aorta, other arteries, organs, and joints in ENPP1 and ABCC6 deficiencies are shown in Fig. 5; these were calculated based on those affected individuals who were assessed [%Yes/(Yes + No)] at specific locations. The prevalence of calcification of individual arteries or organs ranged from 25% to 89% of patients with *ENPP1* or *ABCC6* variants. Overall, the prevalence of calcification was very similar, and location of calcification was not significantly different between ENPP1 and ABCC6 deficiencies, with the exception of joint calcification, which was reported in a significantly higher percentage of patients with ENPP1 deficiency. Methods of imaging assessment of calcification are shown in Supplemental Table S1. There is no significant difference in the frequency of organ complications between ENPP1 and ABCC6 deficiencies, ranging from 35% to 79% (Fig. 5*A*). Prevalence of hearing complications was higher in affected individuals with *ENPP1* variants (28/56 [50.0%]) versus *ABCC6* variants (0/14 [0.0%]; *p* = 0.0004) (Fig. 5*A*). Approximately 50% of patients experienced complications in at least three organ systems (cardiac, pulmonary, gastrointestinal, neurological, or hearing) (Fig. 5*B*); there was no difference between the ENPP1 and ABCC6 cohorts. The most frequently reported signs/symptoms, location of calcification, and organ complications are provided in Supplemental Figs. S1 and S2.

Clinical signs of rickets were reported in 40 of 127 affected individuals (36.7%) in the full data set (Table 1); this prevalence was higher in affected individuals with *ENPP1* variants (36/38 [48.6%]) than in those with *ABCC6* variants (3/18 [16.7%]). Figure 5*C* shows the most frequently reported rickets‐related manifestations. Of the 50 surviving affected individuals with *ENPP1* variants, 34 of 48 assessed (70.1%) developed rickets. Eleven affected individuals were diagnosed with rickets or osteomalacia without a prior GACI or PXE diagnosis, a category designated as ARHR2 (Fig. 1); all had confirmed *ENPP1* variants and median age (range) at diagnosis was 5.4 (2.2–54.5) years. Further assessments revealed seven of 11 (64%) affected individuals had evidence of calcification (three of nine assessed had arterial calcification; three of nine had cardiac valve calcification; two of three had periarticular calcification) and eight of 11 (72%) had organ complications (one of eight had cardiac failure; four of nine had heart valve defect; and four of nine had hearing complications) (data not shown).

## Discussion

This retrospective study is the largest to date of individuals with GACI with or without ARHR2 (*N* = 247), reporting the complications of GACI and hypophosphatemic rickets over a broad age range.^(^ ^4^, ^7^ ^)^ The study found that the clinical spectrum for ENPP1 and ABCC6 deficiencies ranges from high risk of death before 6 months of age, to rickets later in childhood or osteomalacia in adulthood, with a potential for multiorgan involvement. Additionally, this study extensively characterizes infants with *ABCC6* variants, allowing for comparison with infants with *ENPP1* variants. This enabled us to infer overlapping pathomechanisms for ENPP1 and ABCC6 deficiencies.

Overall survival was 45% and age‐dependent, with prenatal death accounting for 24% of total deaths. Risk of mortality was limited to primarily the first 6 months, with the highest risk just after birth. Of the affected individuals in the full data set, five died after 1 year of age (range, 1.5–12.5 years) due to cardiac (*n* = 3) and neurological (*n* = 2) complications. This represents 7% of affected individuals who survived their first year of life (*n* = 71), which suggests a continued risk of mortality at a young age despite surviving the “critical period” of first 6 months.

At first glance, the minimal data set demonstrated a substantial higher mortality compared to the full data set. However, the difference seems to be driven in part by the higher capture of prenatal deaths (minimal, 19.2%, versus full, 7.9%). When prenatal mortality is removed, the number of deaths is similar between both data sets, which suggests that the full data set is representative of the population.

High prenatal and early life mortality is coupled with extensive arterial calcification due to low PP_i_ levels. It stands to reason that initiation of bisphosphonates, structural analogs of PP_i_ with high affinity for hydroxyapatite, could improve survival by exogenously compensating for the low PP_i_ levels in infancy. Bisphosphonates consist of a core nonhydrolyzable carbon P‐C‐P motif that create a more stable (less hydrolyzable) PP_i_ analogue. Etidronate, the first‐generation bisphosphonate, binds directly to hydroxyapatite with high affinity, resulting in inhibition of bone mineralization. The second and third generation bisphosphonates (nitrogen‐containing) cause osteoclast apoptosis and limit bone resorption.^(^ ^21^ ^)^ Bisphosphonates have been used to treat GACI with varying degrees of success.^(^ ^4^, ^5^, ^6^ ^)^ A previous study reported 65% survival of GACI patients receiving bisphosphonates compared to 31% of patients who did not (*p* = 0.026).^(^ ^4^ ^)^ However, our study found that age of initiation of bisphosphonates varied over the critical period of high mortality, and some patients initiated bisphosphonates after the critical period, when the risk of mortality is much lower. Thus, the start‐time matched analysis is an appropriate approach to account for the high rate of death and varied age of bisphosphonate initiation. The results demonstrated no significant benefit in using bisphosphonate. We note a lack of overlap between the confidence intervals around the age of 1 month; however, we caution to not interpret this as validation of bisphosphonate benefit within 1 month. A Kaplan‐Meier survival analysis with either etidronate or other bisphosphonates initiated within 6 months of age versus no bisphosphonate treatment found no survival benefit of either cohort if initiated after 7 days of age. Etidronate has been considered the more appropriate option among bisphosphonates, because the goal is to inhibit bone mineralization (as opposed to bone resorption). However, neither etidronate nor other bisphosphonates showed significant differences in the survival curves if initiated after the first week of life. This might suggest that the class of bisphosphonates does not make a difference at an early age. An alternative possibility is that bisphosphonates have no real impact in survival, and that there is a selection bias of which patients (ie, less severe) are treated with bisphosphonates. However, we could not assess for possible variables that account for healthier affected individuals receiving bisphosphonate treatment, or could not account for possible different dose strength and frequency. A prospective study to evaluate bisphosphonate benefit would be required to demonstrate if prenatal or early postnatal initiation provides a benefit. Although our analysis did not show that the use of bisphosphonates in infancy would be beneficial after 7 days of age, it also did not show it would be detrimental to survival. Thus, the decision to use bisphosphonate continues to be at the discretion of the physician and family.

Similar calcification profiles observed in ENPP1 and ABCC6 deficiencies suggest a common pathological mechanism underlying ectopic calcification. There was a very close overlap of frequency and distribution of calcified arteries and aorta between the ENPP1 and ABCC6 cohorts, including the number of arterial beds with calcification found between the two groups. These findings suggest that both ENPP1 and ABCC6 play a role in ectopic calcification, most likely through an overlapping pathway regulating PP_i_. Patients with *ENPP1* variants have very low to barely detectable PP_i_ levels; this has been associated with early onset of arterial calcification.^(^ ^11^ ^)^ Similarly, adult patients with ABCC6 deficiency and PXE diagnosis exhibited ~50% reduction in plasma PP_i_ levels; consistent findings were reported in *Abcc6*‐mutated mouse models.^(^ ^12^, ^13^ ^)^ It is not clear why some patients with *ABCC6* variants present with arterial calcification at infancy, whereas others present with PXE as adults. It is possible that the PP_i_ levels are extremely low in this ABCC6‐deficient infant population, similar to ENPP1‐deficient patients, which might explain the high risk of calcification of arteries and organs similar to infants with ENPP1 deficiency. However, plasma PP_i_ levels in infants with *ABCC6* variants have not been reported. No genotype phenotype correlation has been identified for either ENPP1 or ABCC6 deficiencies, suggesting variable expressivity of both *ENPP1* and *ABCC6* variants. Identical pathogenic variants in *ABCC6* or *ENPP1* have been shown to cause either PXE or GACI in different patients.^(^ ^7^ ^)^ For example, an identical homozygous pathogenic variant in *ENPP1* led to one family member presenting with ARHR2 without arterial calcification and the second family member presenting with arterial calcification and hypophosphatemia.^(^ ^17^ ^)^ This suggests that genetic modifiers play a role in the presenting phenotype. PP_i_ levels are regulated by ABCC6, ENPP1 (hydrolyzing adenosine triphosphate [ATP] to PP_i_ and adenosine monophosphate [AMP]) and tissue‐nonspecific alkaline phosphatase (TNAP), which degrades PP_i_ into inorganic phosphate.^(^ ^8^, ^10^, ^12^, ^22^ ^)^ Additionally, CD73 hydrolyzes AMP to adenosine, which also influences TNAP activity.^(^ ^22^ ^)^ Genetic modifications or environmental influences to ENPP1, TNAP, or CD73 expression or function may contribute to setting the pathogenic levels of PP_i_ in ABCC6‐deficient infants.

A trend of higher prevalence of neurological complications was observed in affected individuals with *ABCC6* versus *ENPP1* variants. Seizures or epilepsy were reported in 13% and 21% of our ENPP1 and ABCC6 cohorts, respectively. Cumulative incidence of epilepsy in the general population is 0.66% at age 10 years, suggesting *ENPP1* and *ABCC6* variants significantly increase seizure risk.^(^ ^23^ ^)^ Stroke has been recognized as a complication in patients with ENPP1 deficiency, but this was not based on systematic analyses. The prevalence of stroke in a Dutch adult PXE cohort (*n* = 178) was higher (8%) versus a general population matched for age (3%).^(^ ^24^ ^)^ Our study reports a prevalence of stroke in an ABCC6‐deficient pediatric cohort. Thus, this study highlights the broad range of neurological complications in both ENPP1 and ABCC6 deficiencies, likely consequences of calcification and stenosis in intracranial arteries.

Despite the similar calcification phenotype between ENPP1 and ABCC6 deficiencies, there was a disparity in the mortality rate and the presence of rickets in the ENPP1 versus ABCC6 cohorts. The risk of mortality was low in *ABCC6* variants relative to *ENPP1* variants. It is interesting to note that cardiovascular complications tend to be the cause of death in individuals with GACI yet affected individuals with *ABCC6* variants show similar or higher prevalence of cardiovascular complications compared to *ENPP1*. The factors leading to mortality of individuals with GACI are unknown.

Of the ENPP1‐deficient affected individuals that survived the initial critical period, 70% reported signs of rickets. This is in contrast to the three of 18 (17%) with *ABCC6* variants. Further analysis of the rickets observed in the three ABCC6‐deficient affected individuals disclosed that it was not accompanied by hypophosphatemia and hyperphosphaturia and was likely a consequence of etidronate administration. It is worth noting that rickets has not been reported in ABCC6‐deficient adults diagnosed with PXE. This study also identified an ENPP1‐deficient population without GACI diagnosis with rickets (ARHR2). Although these cases did not carry a GACI diagnosis, ~70% demonstrated evidence of arterial or organ calcification, symptoms of hearing loss, or cardiovascular complications similar to those seen in survivors of GACI. Together these data show that GACI and rickets (ARHR2) represent a spectrum of ENPP1 deficiency.

It is not clear why there is an overlapping GACI phenotype (arterial calcification with cardiovascular complications) between ENPP1 and ABCC6 deficiencies but a difference in the eventual development of rickets with *ENPP1* variants. ENPP1 deficiency is associated with elevated FGF23, a bone‐derived hormone that regulates renal phosphate reabsorption and vitamin D metabolism.^(^ ^25^, ^26^, ^27^ ^)^ Elevated levels of FGF23 result in phosphate wasting and hypophosphatemic rickets.^(^ ^28^, ^29^ ^)^ The mechanism underlying elevated FGF23 levels in ENPP1 deficiency has not been elucidated, but it is notable that FGF23 is largely secreted by osteocytes and osteoblasts, where ENPP1 is expressed.^(^ ^26^ ^)^ *Enpp1* −/− knockout mice show a 12‐fold greater expression of *Fgf23* mRNA compared to wild‐type littermates, which suggests that ENPP1 function can regulate osteoblastic cellular expression, including elevating FGF23, and thus inducing hypophosphatemic rickets.^(^ ^30^ ^)^ To our knowledge, elevation of FGF23 has not been reported in ABCC6‐deficient patients. ABCC6 is a transmembrane protein primarily expressed in the liver and kidney, where through an unknown mechanism it exports ATP, among other substrates, into the circulation.^(^ ^8^ ^)^ Membrane‐bound ENPP1 catalyzes the hydrolysis of ATP to AMP, generating PP_i_, which in turn inhibits mineralization.^(^ ^12^, ^31^ ^)^ The functional loss of ABCC6 or ENPP1 thereby results in reduced serum PP_i_ and AMP levels, leading to systemic ectopic calcification as seen in mouse models and patients with PXE and/or GACI.^(^ ^7^, ^11^, ^32^ ^)^

Study limitations are typical of a multicenter retrospective design and include incompleteness/inconsistency in data acquired manually, data limited to available medical records, documentation not systematically recorded for all variables, dates of first observation not reflecting physiologic onset, and lack of standardized assessments. Thus, actual prevalence of clinical manifestations could be higher than what is reported due to incomplete evaluations. Additionally, the number of affected individuals with *ABCC6* variants was small (*n* = 19) versus affected individuals with *ENPP1* variants. Despite the limitations, this study has demonstrated poor survival in infants during the first 6 months, highlighting the need for new therapies to improve survival.

In summary, this is the largest retrospective study to assess the natural history of GACI or ARHR2, including long‐term mortality, and a phenotypic comparison of GACI from ABCC6 versus ENPP1 deficiency. Mortality associated with this disorder is highest in the first few months of life, despite bisphosphonate treatment, and is predominant in ENPP1 deficiency. Patients with ENPP1 or ABCC6 deficiencies can experience morbidity throughout their life due to multiple organ dysfunctions. The findings of this natural history study provide insight into the complexity of the disease and may help develop more effective management strategies or drive identification of new therapeutics to improve patient prognosis.

## Disclosures

CRF reports a collaboration with Inozyme Pharma, Inc. as part of a Cooperative Research and Development Agreement. MEH reports a collaboration with Inozyme Pharma, Inc as part of a Cooperative Research and Development Agreement. MZM received honoraria from Inozyme Pharma, Inc. DS received consulting fees from Inozyme Pharma, Inc. EY is a former employee of Inozyme Pharma, Inc. at the time of the study and owns stock in Inozyme Pharma, Inc. WAG reports a collaboration with Inozyme Pharma, Inc. as part of a Cooperative Research and Development Agreement. RIG received nonsalary research support for studies unrelated to this project from the following companies: Ultragenyx Pharmaceutical, QED Therapeutics, Inc, Calcilytix; she is a medical advisor for GACI Global. QL received salary from Inozyme Pharma, Inc. on contract basis. PH is a former employee of Inozyme Pharma, Inc. at the time of the study and owns stock in Inozyme Pharma, Inc. GK is a full‐time employee of Inozyme Pharma, Inc. and owns stock in Inozyme Pharma, Inc. FR received nonsalary research support for the conduction of the trial as well as consultancy fees from Inozyme Pharma, Inc. KK, UB, YN, and GB have no conflict of interest. All authors have potential conflicts of interests denoted in this section. The authors who have no conflicts of interest to declare are listed as such. Please confirm if more information is required.

## Supporting information


**Supplemental Table S1.** Imaging assessment of calcification at initial presentation in individuals with *ENPP1* or *ABCC6* variants.

**Supplemental Fig. S1.** Prevalence and number of locations for (*A*) Arterial Calcification, (*B*) Aortic Calcification, (*C*) Organ Calcification, and (*D*) Joint Calcification.

**Supplemental Fig. S2.** Prevalence and number of locations for organ involvement: (*A*) Cardiac, (*B*) Neurological, (*C*) Pulmonary, and (*D*) Gastrointestinal.

## Data Availability

The data that support the findings of this study are available from the corresponding authors upon reasonable request.
