# Supplementary material for: Ectopic Calcification and Hypophosphatemic Rickets: Natural History of ENPP1 and ABCC6 Deficiencies
Source: J Bone Miner Res. 2021 Aug 16;36(11):2193–202. doi: 10.1002/jbmr.4418 (PMC8595532; doi:10.1002/jbmr.4418)
Supplement: Supplementary file 1 — Supplemental Table S1. Imaging assessment of calcification at initial presentation in individuals with ENPP1 or ABCC6 variants. [file JBMR-36-2193-s001.docx]

**Supplemental Table 1.** Imaging Assessment of Calcification at Initial Presentation in Individuals With *ENPP1* or *ABCC6* Variants

|  | **Imaging assessment** | | | | | | **Total affected individuals, %** |
| --- | --- | --- | --- | --- | --- | --- | --- |
|  | **Sonography** | **Radiography** | **CT** | **CT angiography** | **MRI** | **Echo** |  |
| **No. of affected individuals** | 84 | 82 | 51 | 19 | 23 | 87 | 101 |
| **Arterial calcification, No. (%)** | 52 (61.9) | 30 (36.6) | 37 (72.5) | 7 (36.8) | -- | 53 (60.9) | 70 (69.3) |
| **Organ calcification, No. (%)** | 28 (33.3) | -- | -- | -- | -- | -- | 28 (33.3) |
| **Joint calcification, No. (%)** | -- | 17 (20.7) | 10 (19.6) | -- | -- | -- | 21 (23.1)^a^ |

Echo, echocardiogram.

^a^ Based on any assessment.

**Figure Legends**

**Supplemental Figure 1.** Prevalence and number of locations for (A) Arterial Calcification, (B) Aortic Calcification, (C) Organ Calcification, and (D) Joint Calcification.

**Supplemental Figure 2.** Prevalence and number of locations for organ involvement: (A) Cardiac, (B) Neurological, (C) Pulmonary, and (D) Gastrointestinal.
