# Supplementary figures and images for: Ectopic Calcification and Hypophosphatemic Rickets: Natural History of ENPP1 and ABCC6 Deficiencies
Source: J Bone Miner Res. 2021 Aug 16;36(11):2193–202. doi: 10.1002/jbmr.4418 (PMC8595532; doi:10.1002/jbmr.4418)

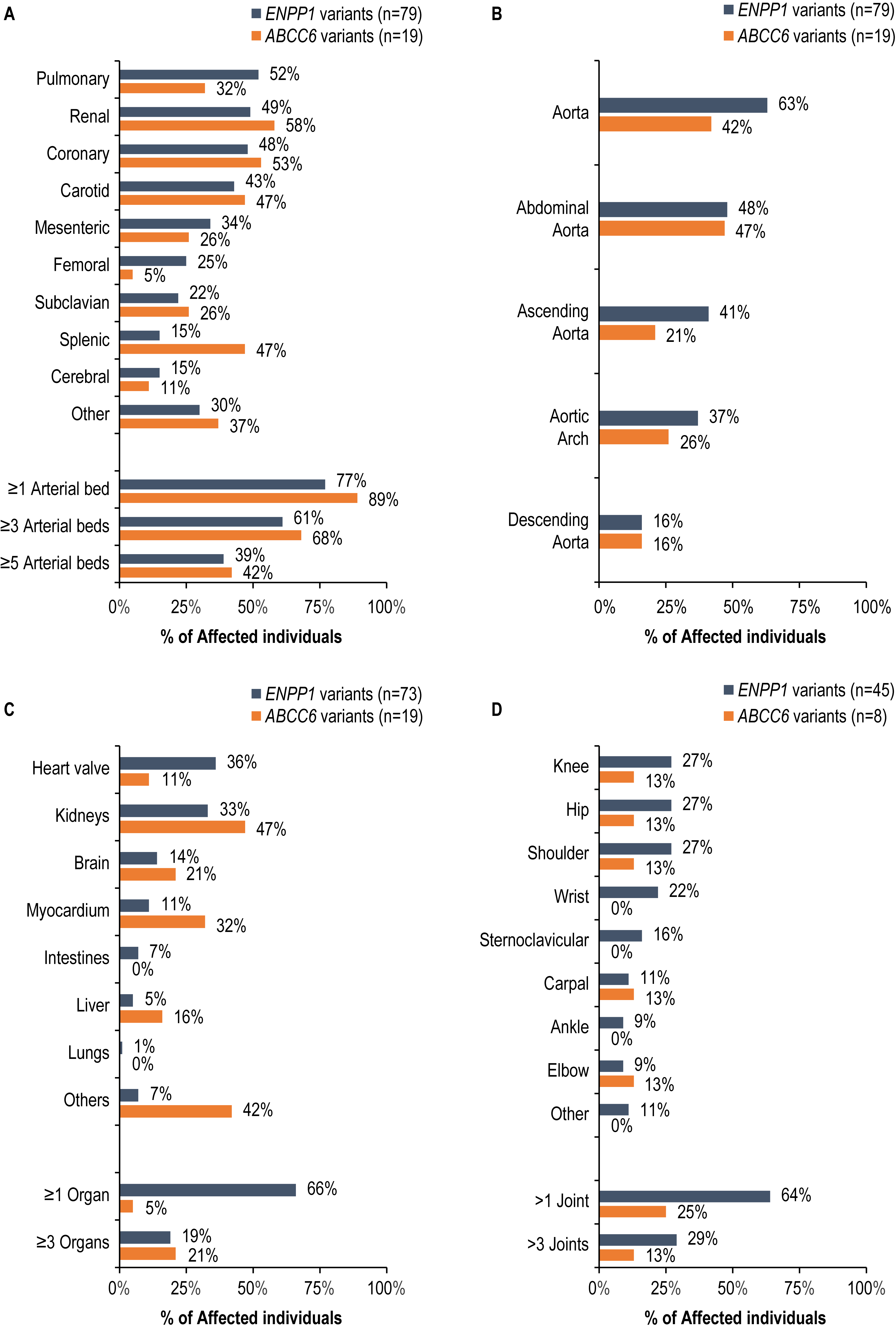

Supplement: Supplementary file 2 — Supplemental Fig. S1. Prevalence and number of locations for (A) Arterial Calcification, (B) Aortic Calcification, (C) Organ Calcification, and (D) Joint Calcification. [file JBMR-36-2193-s003.tif]

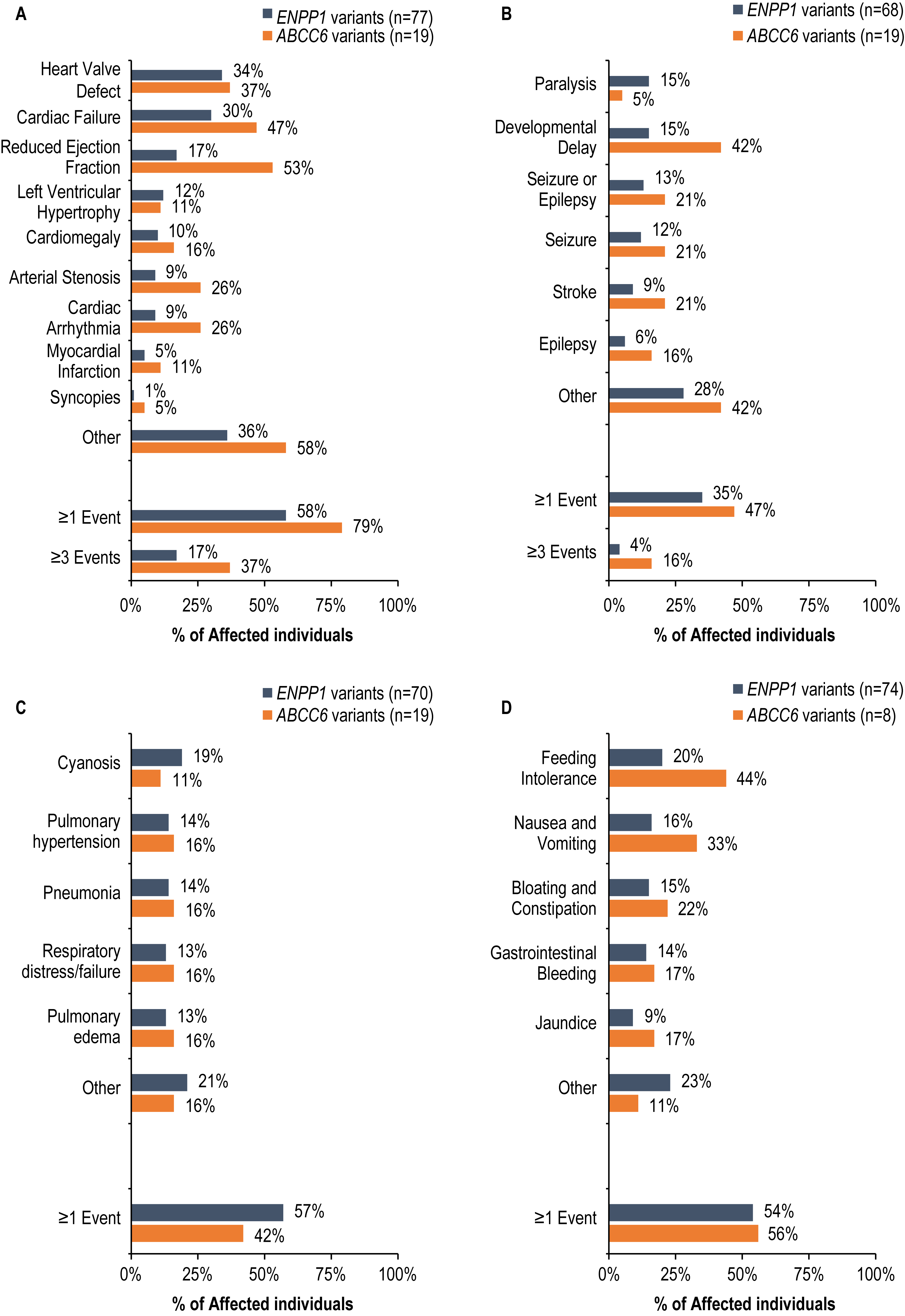

Supplement: Supplementary file 3 — Supplemental Fig. S2. Prevalence and number of locations for organ involvement: (A) Cardiac, (B) Neurological, (C) Pulmonary, and (D) Gastrointestinal. [file JBMR-36-2193-s002.tif]
